# Abnormal cross-frequency coupling in the tinnitus network

**DOI:** 10.3389/fnins.2014.00284

**Published:** 2014-09-25

**Authors:** Ilya Adamchic, Berthold Langguth, Christian Hauptmann, Peter A. Tass

**Affiliations:** ^1^Jülich Research Center, Institute of Neuroscience and Medicine, INM-7, NeuromodulationJülich, Germany; ^2^Department of Psychiatry and Psychotherapy, University of RegensburgRegensburg, Germany; ^3^Interdisciplinary Tinnitus Center, University of RegensburgRegensburg, Germany; ^4^Department of Neurosurgery, Stanford UniversityStanford, CA, USA; ^5^Department of Neuromodulation, University of CologneCologne, Germany

**Keywords:** tinnitus pitch, oscillations, delta band activity, alpha rhythm, gamma band activity, coordinated reset neuromodulation, cross frequency coupling

## Abstract

Neuroimaging studies have identified networks of brain areas and oscillations associated with tinnitus perception. However, how these regions relate to perceptual characteristics of tinnitus, and how oscillations in various frequency bands are associated with communications within the tinnitus network is still incompletely understood. Recent evidence suggests that apart from changes of the tinnitus severity the changes of tinnitus dominant pitch also have modulating effect on the underlying neuronal activity in a number of brain areas within the tinnitus network. Therefore, in a re-analysis of an existing dataset, we sought to determine how the oscillations in the tinnitus network in the various frequency bands interact. We also investigate how changes of tinnitus loudness, annoyance and pitch affect cross-frequency interaction both within and between nodes of the tinnitus network. Results of this study provide, to our knowledge, the first evidence that in tinnitus patients, aside from the previously described changes of oscillatory activity, there are also changes of cross-frequency coupling (CFC); phase-amplitude CFC was increased in tinnitus patients within the auditory cortex and the dorsolateral prefrontal regions between the phase of delta-theta and the amplitude of gamma oscillations (Modulation Index [MI] 0.17 in tinnitus patients vs. 0.08 in tinnitus free controls). Moreover, theta phase in the anterior cingulate region modulated gamma in the auditory (MI 0.1) and dorsolateral prefrontal regions (MI 0.19). Reduction of tinnitus severity after acoustic coordinated reset therapy led to a partial normalization of abnormal CFC. Also treatment induced changes in tinnitus pitch significantly modulated changes in CFC. Thus, tinnitus perception is associated with a more pronounced CFC within and between nodes of the tinnitus network. CFC can coordinate tinnitus-relevant activity in the tinnitus network providing a mechanism for effective communication between nodes of this network.

## Introduction

Phantom auditory perception refers to the conscious awareness of an internally generated sensory percept when no matching auditory stimulus is present (Eggermont, 2003; Snow, 2004; Møller et al., 2010). Synchronization between different cortical areas in the tinnitus network plays an important role in the pathophysiological basis of tinnitus (Schlee et al., 2008, 2009). Synchronization at single frequencies between distant tinnitus hubs is thought to be relevant to how these regions interact within the tinnitus network, and such interactions have been well studied (Schlee et al., 2008, 2009; Vanneste et al., 2011; Silchenko et al., 2013). However, it remains unclear how different frequency bands interact both within and between various nodes of the tinnitus network. Compared to the synchronization at single frequencies cross-frequency coupling (CFC) would allow for much more complex corticocortical interactions and has also been extensively studied in various brain signals and across distant cortical regions (Shils et al., 1996; Schack et al., 2002; Buzsaki and Draguhn, 2004; Palva et al., 2005; Canolty et al., 2006; Palva and Palva, 2007; Darvas et al., 2009). CFC in the brain suggests that non-linear interactions can play a crucial role in the coordination of complex cortical computation. Here, we investigate cross-frequency phase-amplitude coupling both within and between hubs of the tinnitus network, using a comodulation analysis introduced by Tort et al. (2008). Previous encephalographic (EEG), magnetoencephalographic (MEG) and electrocorticographic studies have demonstrated the possibility of high frequencies (e.g., in the gamma band) being modulated by lower frequencies (Mormann et al., 2005; Canolty et al., 2006; Osipova et al., 2008). For example, it has been shown that high-frequency gamma power (30–70 Hz) is phase-locked to alpha oscillations (8–13 Hz) in the ongoing MEG signals (Osipova et al., 2008). Another study showed that theta phase modulates gamma (30–50 Hz) amplitude (Lakatos et al., 2005). Therefore, we focused our analysis on cross-frequency phase-amplitude interactions between frequencies ranging from 1 to 30 Hz, covering the delta, theta, alpha and beta bands, with frequencies from 30 to 48 Hz, i.e., gamma band.

The experience of tinnitus involves sensory and emotional components that are partially interdependent (Jastreboff, 1990; Weisz et al., 2005; De Ridder et al., 2011a). Recently there has been a growing interest in how the separate psychoacoustic characteristics of the tinnitus signal (e.g., loudness, pitch, noise vs. pure tone) are encoded and how their changes affect neuronal processing in the tinnitus aversive network (van der Loo et al., 2009; Vanneste et al., 2010; Adamchic et al., 2012a). Traditionally therapeutic interventions have been evaluated either in their effects on tinnitus distress or on tinnitus loudness (Aazh et al., 2008; De Ridder and Vanneste, 2011; Landgrebe et al., 2012; Tass et al., 2012). However, recent results suggest existence of an interdependence between perceptual characteristics of tinnitus and the related handicap. In addition to reduction of subjective tinnitus loudness that has been shown to have a strongly influence on tinnitus distress, changes in other characteristics like tinnitus pitch may have an important modulating effect as well (Bartels et al., 2007; Adamchic et al., 2012a; Tass et al., 2012). In a recent article, we reported that reduction of both subjectively perceived tinnitus loudness and tinnitus annoyance after acoustic CR neuromodulation (Tass, 2003; Tass and Popovych, 2012; Tass et al., 2012) significantly correlated with the modulus of the tinnitus pitch ratio (Adamchic et al., 2012a). In order to identify the neuronal correlates of the pitch change we divided patients into two groups: (i) a group with pronounced pitch change (PC), (ii) a group with no or minimal tinnitus pitch change (NPC), while keeping the relief of subjectively perceived tinnitus loudness and annoyance similar in both groups. Patients in the PC group had modulus pitch change ratio, that is, the absolute value of the pitch change ratio, of <0.3 and a reduction of VAS loudness (VAS-L)/VAS annoyance (VAS-A) of −26.9[17.5]/−25.7[15.2] [mean, (standard deviation)]. Patients in the NPC group had a modulus pitch change ratio of >0.3 and reduction of VAS-L/VAS-A of −27.1[10.3]/−26.2[12.1]. In patients with pronounced tinnitus pitch change there were significantly stronger treatment related changes of brain oscillatory activity in a distributed network of brain areas which included the left superior temporal cortex (AC), the dorsolateral prefrontal region (DLPFC), the left premotor cortex, and the anterior cingulate cortex (ACC). Changes of brain activity in the region of the superior temporal gyrus (Brodmann area 22; BA) in the gamma band correlated with the tinnitus pitch change ratio. These findings suggest that changes of different perceptual characteristics of tinnitus (pitch and loudness) may have modulating effect on each other, on tinnitus annoyance and on the underlying neuronal activity (Adamchic et al., 2012a) and fits with the hypothesis that tinnitus results from pathological oscillatory activity in various dynamic overlapping brain networks (Schlee et al., 2009; De Ridder et al., 2011a, 2014; Vanneste et al., 2011; Langguth et al., 2013; Silchenko et al., 2013). This finding also showed that a synchronous tinnitus related linkage is possible between DLPFC, AC and ACC, but the functioning of such linkage was not readily apparent. Therefore, in this study we investigated cross-frequency phase-amplitude coupling both within and between cortical areas that were previously shown to be part of the tinnitus network. Firstly we compared CFC in tinnitus patients and healthy controls and secondly we investigated the effects of treatment induced changes of the tinnitus loudness, annoyance and pitch.

We used high density EEG data recorded from 32 patients with chronic tonal tinnitus and tested for the existence of phase-amplitude CFC within as well as between three structures: (1) auditory cortex region, (2) dorsolateral-prefrontal region, and (3) anterior cingulate cortex. We hypothesized that tinnitus patients as compared to healthy controls show evidence of abnormal CFC across a broad range of frequencies, and that this CFC is changed by CR neuromodulation induced reduction of the tinnitus symptoms.

## Methods

### Subjects

In this study we re-analyzed EEG data recorded in tinnitus patients who participated in a multicentric randomized, controlled clinical trial on acoustic CR neuromodulation in the treatment of chronic subjective tonal tinnitus [“RESET study,” ClinicalTrials.gov Identifier: NCT00927121 (Tass et al., 2012)]. Inclusion criteria, selection of patients and definition of the PC and NPC groups are described in detail in Adamchic et al. (2012a) and Tass et al. (2012). The baseline VAS-L/VAS-A in all 32 patients from both PC and NPC groups was 68.4[15.3]/66.3[17.5], their average age was 49.6[11.9] years and the pure-tone averages (the average hearing threshold level) of 1–4 and 6–12 kHz were 17.6[9.4] and 39.1[17.5], respectively (Adamchic et al., 2012a). The control group consisted of 16 healthy tinnitus-free subjects (10 men and 6 women). Their average age was 45.0[12.5] years and the pure-tone averages of 1–4 and 6–12 kHz were 12.73[13.69] and 24.29[19.05], respectively.

### CR treatment

In the Tass et al. (2012) study patients were stimulated with acoustic CR neuromodulation for 12 weeks using a portable acoustic device and comfortable earphones. Each patient underwent two EEG recording sessions, the first one on the first treatment day before start of the treatment and the second at the 12-weeks follow up. EEG was recorded at a minimum 2-h break from stimulation. Subjectively perceived tinnitus loudness and tinnitus annoyance were assessed off-stimulation using a VAS-L and VAS-A (Adamchic et al., 2012b). The CR neuromodulation resulted in a significant and clinically relevant decrease of tinnitus severity as measured by VAS-L/VAS-A and TQ scores (Adamchic et al., 2012b,c; Tass et al., 2012).

### EEG data acquisition and data analysis

EEG recordings were obtained in a dimly lit room in a Faraday cage from 128 surface electrodes (HydroCel Geodesic Sensor Net) referenced to Cz. The EEG signals were amplified with a Net Amps 200 amplifier (Electrical Geodesis Inc, Eugene, USA), digitized at 1 kHz and band-pass filtered from 0.1 to 400 Hz. Recordings were made in an awake state with the subjects in alternating 2 min intervals with eyes closed and with eyes open and the eyes closed data was selected for further analysis. Each EEG recording was corrected for eye blink and movement artifacts using the surrogate model approach in BESA (Brain Electrical Source Analysis, MEGIS Software, 5.2 version) (Ille et al., 2002). Surface EEG was transformed into brain source activity using the source montage approach in BESA (Scherg et al., 2002). A source model was generated with regional neural sources placed in the regions of interest (ROI). Based on the results of Adamchic et al. (2012a), the source montage consisted of ROIs with sources placed in the regions of the left auditory cortex (AC), right dorsolateral-prefrontal cortex (DLPFC) and anterior cingulate cortex (ACC). Talairach coordinates of these ROIs were: AC (x −58, y −45, z 16), DLPFC (x 53, y 14, z 32), ACC (x 6, y 37, z 11). Additional probe-sources were placed into the occipital lobe, orbito-frontal region, into the area of the central sulcus in both hemispheres as well as into the left DLPFC and right AC. These sources outside the ROIs acted as a spatial filter and reduced the contribution of these regions to the ROI.

EEG signals were adaptively filtered using the data-driven empirical mode decomposition (EMD) approach where the basis functions are derived directly from the time-series itself (Huang et al., 1998). EMD provides a set of intrinsic mode functions (IMF) for signals from each source with each IMF corresponding to one of the co-existing time scales. EMD allows avoidance of drawbacks caused by the band pass filtering (Florin et al., 2010). Phases were extracted by Hilbert transformation for every IMF from 1 to 30 Hz. The amplitude for high frequencies between 30 and 48 Hz was calculated using Hilbert transformation for each 1 Hz frequency step (4 Hz wide band pass filter). Frequency bands were defined as follows: delta (1–4 Hz), theta (4–8 Hz), alpha (8–13 Hz), gamma (30–48 Hz).

### Estimation of phase-amplitude coupling

Given a set of IMFs for each of the source signals, we used the modulation index (MI) and comodulation analysis introduced by Tort et al. (2008) to calculate hierarchical phase-amplitude coupling (Lakatos et al., 2005; Tort et al., 2008, 2010). The MI represents a normalized measure of CFC between 2 frequency ranges of interest: a phase-modulating lower frequency (*fp*) and an amplitude-modulated (*fa*) higher frequency. To calculate the MI the phases of phase-modulating oscillation between 0 and 360° were binned into eighteen 20° wide bins and the mean amplitude of *fa* associated to each 20° wide bin was computed. Then the mean amplitude in each bin was normalized by dividing each bin's value by the sum of all 18 bins, resulting in a phase-amplitude function. A uniform phase-amplitude distribution, that is, the *fa* amplitude is the same for all *fp* phase bins, indicates the absence of phase-amplitude coupling. The higher the coupling between *fa* amplitude and *fp* phase, the further away from the uniform distribution the phase-amplitude distribution will get. The distance between the two distributions P and Q can be defined as $D_{KL}\left(P,Q\right)=\sum_{j\;=\;1}^{N}P(j)\log\;\left(\frac{P(j)}{Q(j)}\right)$ where N = 18 is the number of phase bins, j is a number of the given bin (Tort et al., 2008, 2010). The modulation index (MI) is given by $\text{MI}=\frac{D_{KL}(P,Q)}{log(N)}$ which is a measure between 0 (no phase-amplitude modulation) and 1 (maximal phase-amplitude modulation) (Tort et al., 2008, 2010).

### Estimation of phase synchronization and coherence

Coherence between EEG signals was calculated for every patient separately using IMF for which significant CFC was found. For example, when in a particular patient the CFC was found between ACC IMF phase with frequency 3.5 Hz and AC gamma amplitude with frequency 35 Hz, then single frequency coherence and phase synchronization (PS) were also calculated in this particular patient between all three tested ROI (i.e., ACC, AC, and DLPFC) for the IMF in delta (3.5 Hz) and gamma (35 Hz). Coherence was calculated using the standard MATLAB function “mscohere,” subsequently averaging across all epochs of a given patient (1.5-s sliding window). To calculate PS continuously over time rather than across trial repetitions, the phase synchronization (Tass et al., 1998; Rosenblum et al., 2001)—also called phase locking value (PLV) (Lachaux et al., 1999)—was defined by $\text{PS}=\frac{1}{N}\left|\sum_{n\;=\;1}^{N}\;exp\;(j\theta (source_{x},source_{y}{}_{,}\text{n}))\right|$ where θ (*source*_*x*_, *source*_*y*_, n) is the phase difference between 2 source signals ϕ1(*source_x_*, n)—ϕ2(*source_y_*, n) and N is the number of time points in the analyzed signal. PS measures the variability of the phase difference between two signals over a certain time interval and reflects the consistency of phase lag between them. To this end, PS detects whether there is one prominent peak in the distribution of the phase difference (Tass et al., 1998; Rosenblum et al., 2001). If the phase difference varies little, PS is close to 1; otherwise it is close to zero.

### Statistical analysis

In the current study, interactions between changes of MI in PC and NPC groups were investigated using repeated measure analysis of variance (rmANOVA). MIs at the 12 weeks visit were compared to the baseline values using paired samples *t*-test. Comparisons between MIs in tinnitus patients and healthy controls were performed using *t*-tests for independent groups. Pearson correlation coefficients were calculated to study whether the changes of MI correlated with changes in tinnitus pitch change ratio, VAS-L and VAS-A. Pearson correlation was also performed between changes of MI, PS and coherence. In this study we only use the off-stimulation VAS scores, because EEG recordings were performed off-stimulation. The data are presented as mean [standard deviation]. Results of the statistical tests were corrected for the number of tests conducted using the false discovery rate method (FDR) (Benjamini and Hochberg, 1995).

## Results

### Cross-frequency interactions within and between ROIs

At the 12 weeks visit VAS-L/VAS-A in all 32 patients were reduced by 43.3/44.8% respectively. In the NPC and PC groups VAS-L/VAS-A decreased respectively by 44.2/43.4% and 42.4/44.2%. In this study we only use the off-stimulation VAS scores, because EEG recordings were performed off-stimulation. Modulus of the pitch change ratio was 0.336 in all 32 patients and 0.151/0.521 in the NPC and PC groups respectively.

**Figure 1:**
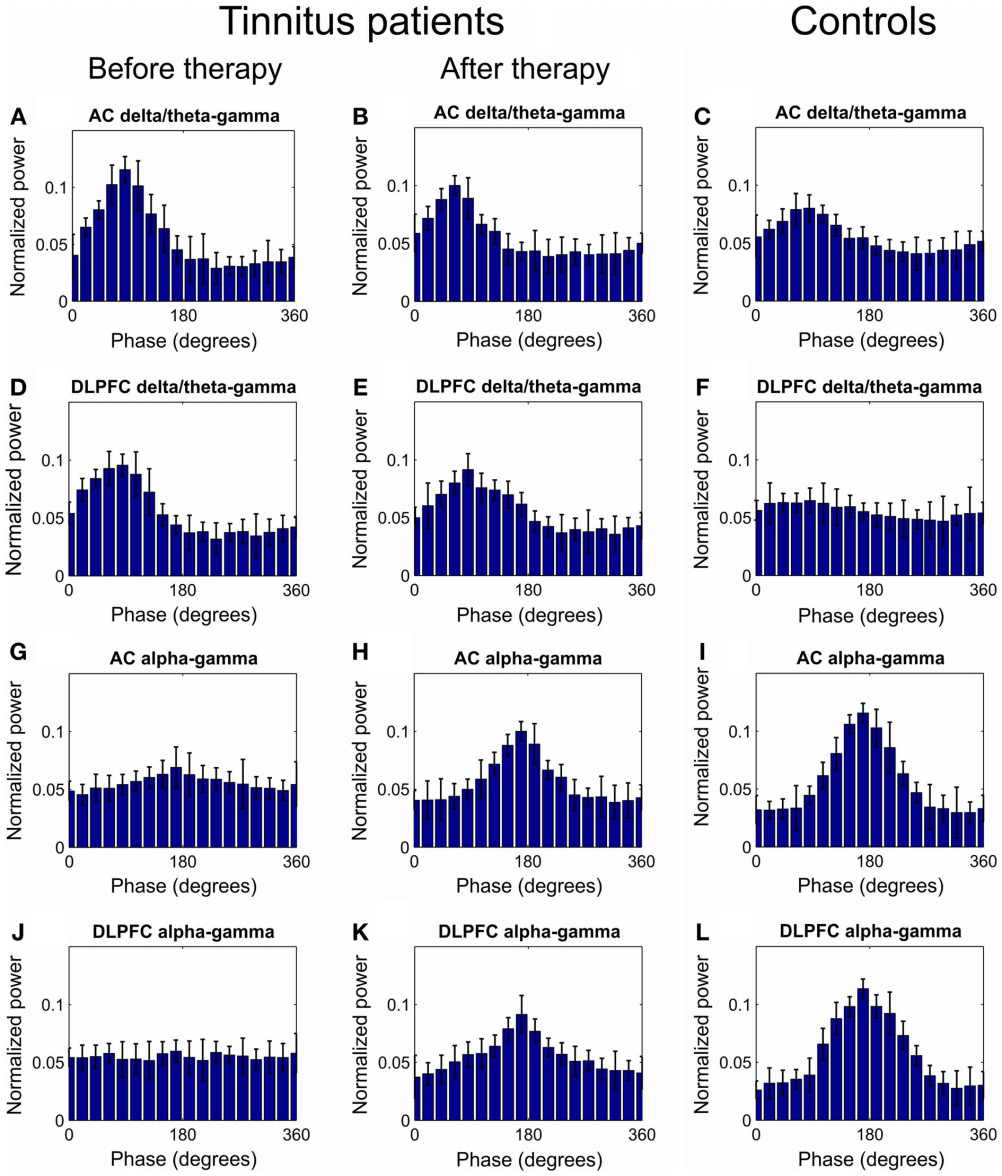
**Grand average of the distribution of gamma power across all delta/theta and alpha phases**. **(A–C)** Distribution of AC gamma power across delta/theta phases at baseline **(A)** and at 12 weeks visit **(B)** in tinnitus patients and in tinnitus free controls **(C)**. **(D–F)** Distribution of DLPFC gamma power across all delta/theta phases at baseline **(D)** and at 12 weeks visit **(E)** in tinnitus patients and in tinnitus free controls **(F)**. **(G,H)** Distribution of AC gamma power across all alpha phases at baseline **(G)** and at 12 weeks visit **(H)** in tinnitus patients and in tinnitus free controls **(I)**. **(J,K)** Distribution of DLPFC gamma power across all alpha phases at baseline **(G)** and at 12 weeks visit **(H)** in tinnitus patients and in tinnitus free controls **(L)**.

**Figure 2:**
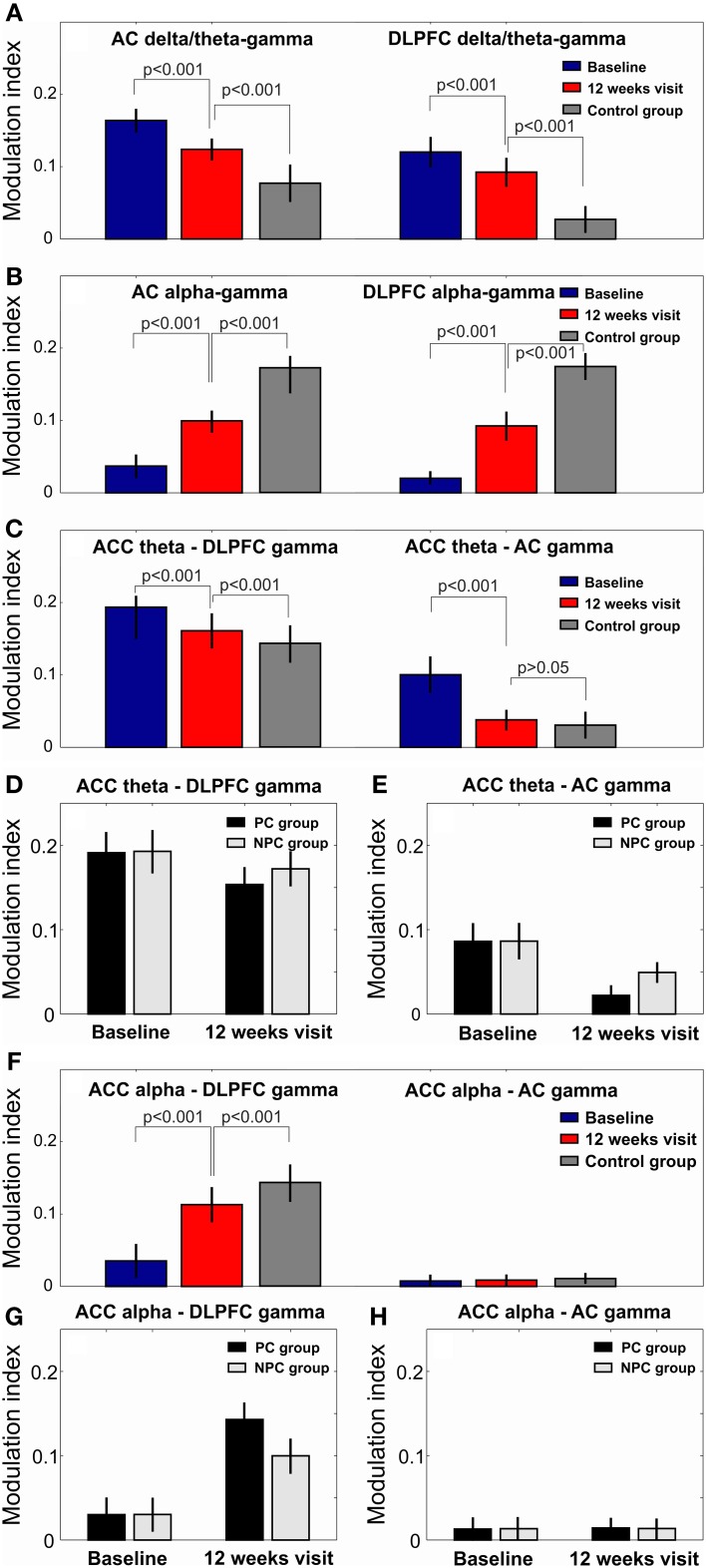
**The modulation index values in all *n* = 32 investigated patients at baseline and at the 12 weeks visit**. The gamma amplitude delta/theta phase modulation index in the AC and DLPFC **(A)**. The gamma amplitude alpha phase modulation index in the AC and DLPFC **(B)**. After 12 weeks of acoustic CR neuromodulation the CFC between delta/theta phase and gamma amplitude approached the CFC in the healthy control group **(A,B)**. CFC between ACC theta phase and DLPFC/AC gamma amplitude **(C)**. The modulation index values for CFC between ACC theta phase and DLPFC/AC gamma amplitude in both PC (*n* = 16) and NPC (*n* = 16) groups at baseline and at 12 weeks visit **(D,E)**. The reduction of CFC between ACC theta phase and DLPFC/AC gamma amplitude was significantly more pronounced in the PC group than in the NPC group at 12 weeks visit as compared to baseline **(D,E)**. CFC between ACC alpha phase and DLPFC/AC gamma amplitude **(F)**. The increase of CFC between ACC alpha phase and DLPFC gamma amplitude was significantly more pronounced in the PC group than in the NPC group at 12 weeks visit as compared to baseline **(G,H)**.

As a first step, we compared the levels of hierarchical phase-amplitude coupling in EEG recordings of the tinnitus patients and healthy controls. At baseline, the comodulation analysis revealed the existence of increased (as compared to the healthy controls) phase-amplitude coupling in the tinnitus patients in the AC and DLPFC between the phase of the delta-theta IMFs (mean IMF frequencies for AC: 3.53[0.79] Hz and DLPFC: 3.21[0.90 Hz]) and the amplitude of the gamma oscillations (AC: 34.84[2.85] and DLPFC: 36.00[1.59] Hz; Figures 1A,C,D,F, 2A). Decreased CFC was found in tinnitus patients as compared to healthy controls in the AC and DLPFC between the phase of the alpha IMFs (mean IMF frequency for AC: 10.19[0.76] and DLPFC: 10.61[0.69] Hz) and the amplitude of the gamma oscillations (AC: 35.81[2.43] and DLPFC: 36.20[3.39] Hz; Figures 1G,I,J,L, 2B). At the 12-week follow up these CFCs in tinnitus patients were significantly modified and approached the levels of the control group (Figures 1, 2A,B).

**Figure 3:**
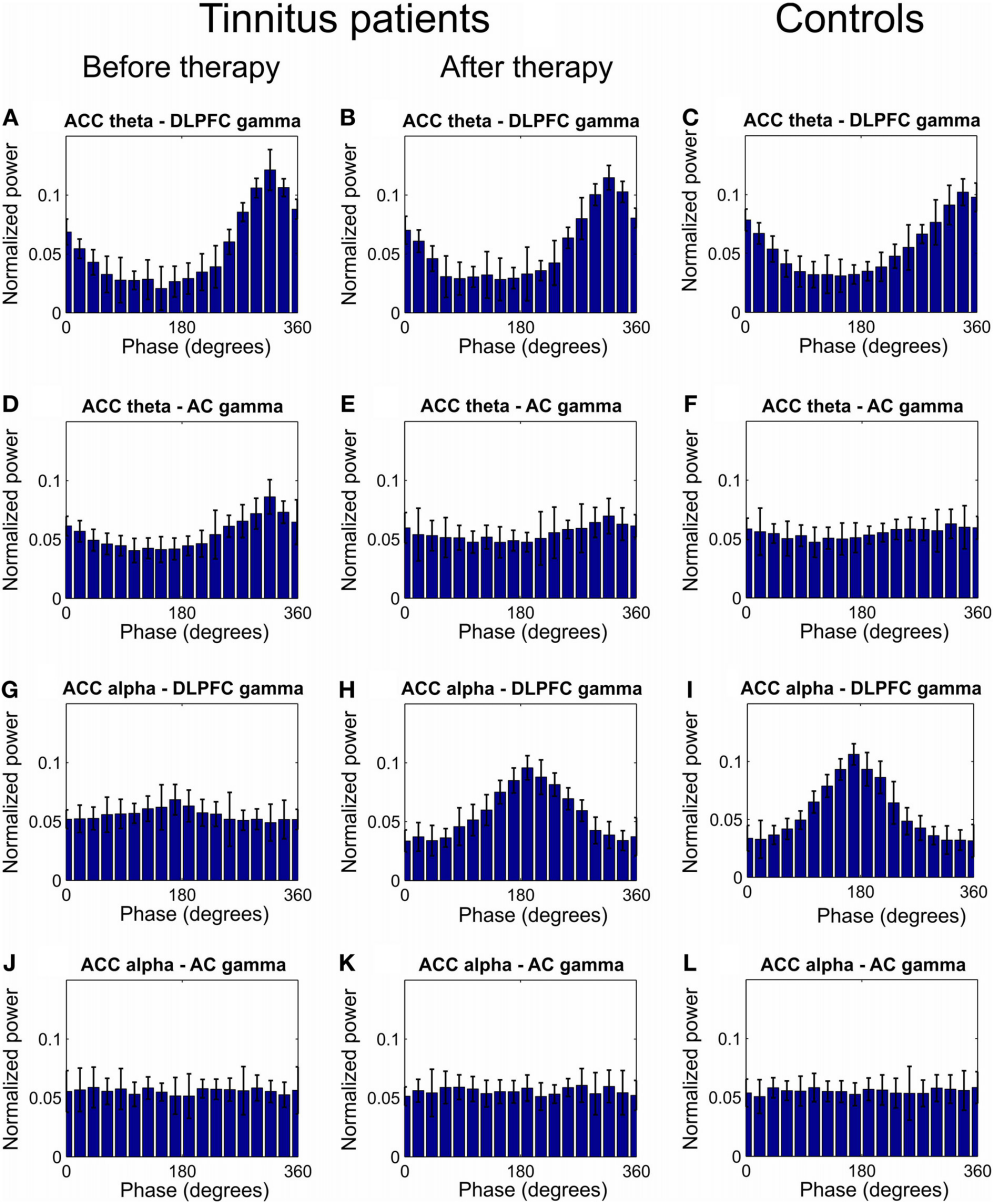
**Grand average of the distribution of gamma power across all ACC theta and alpha phases**. **(A–C)** Distribution of DLPFC gamma power across all theta phases of ACC theta at baseline **(A)** and at 12 weeks visit **(B)** in tinnitus patients and in tinnitus free controls **(C)**. **(D–F)** Distribution of AC gamma power across all theta phases of ACC theta at baseline **(D)** and at 12 weeks visit **(E)** in tinnitus patients and in tinnitus free controls **(F)**. **(G,H)** Distribution of DLPFC gamma power across all ACC alpha phases at baseline **(G)** and at 12 weeks visit **(H)** in tinnitus patients and in tinnitus free controls **(I)**. **(J–L)** Distribution of AC gamma power across all alpha phases in ACC at baseline **(G)** and at 12 weeks visit **(H)** in tinnitus patients and in tinnitus free controls **(L)**.

Further we investigated CFC between phases of the lower frequency modes (delta, theta and alpha) in ACC and amplitudes of the gamma oscillations in the AC and DLPFC. CFC in tinnitus patients as compared to the healthy controls was increased between ACC theta phase (IMF frequency 5.85[0.76] Hz) and the amplitude of the gamma oscillation in the DLPFC (IMF frequency 36.67[2.54] Hz) and AC (IMF frequency 36.61[2.0] Hz; Figures 2C, 3A,C,D,F). This CFC averaged over all of the 32 patients was significantly reduced at the 12-week follow up, as compared to the baseline, between the ACC and DLPFC (MI change −0.022, *p* < 0.001) and between the ACC and AC (MI change −0.062, *p* < 0.001; Figures 3A–F).

We next investigated whether there was an interaction between MI changes after 12 weeks of CR neuromodulation in the PC and NPC groups. The rmANOVA revealed a significant interaction (*p* < 0.05) between ACC theta—DLPFC gamma MI changes in the PC and NPC groups. Specifically, ACC theta—DLPFC gamma MI reduced significantly greater in the PC group (MI change −0.033, *p* < 0.001) as compared to the NPC group (MI change −0.010, *p* < 0.001; Figure 2D). Significant interaction was also revealed for the ACC theta—AC gamma CFC with respect to group (*p* < 0.05). The reduction of MI in the PC group was significantly more pronounced (MI change −0.074, *p* < 0.001) as compared to the NPC group (MI change −0.050, *p* < 0.001; Figure 2E).

At baseline there was a decreased CFC between the ACC alpha phase and the amplitude of the gamma oscillations in the DLPFC (Figures 2F, 3G,I) but not between the ACC alpha and the AC gamma (Figures 2F, 3J,L) in tinnitus patients as compared to the healthy controls. At the 12 weeks follow up, these CFCs averaged over all of the 32 tinnitus patients were MI 0.120 (MI change 0.085, *p* < 0.001) between the ACC and the DLPFC (Figures 2F, 3H) and MI 0.009 (MI change 0.001, *p* > 0.05) between the ACC and AC (Figures 2F, 3K). The rmANOVA revealed a significant interaction (*p* < 0.05) between MI changes with respect to group, that is, ACC alpha—DLPFC gamma phase-amplitude coupling increased significantly more pronounced in the PC group (MI change 0.106, *p* < 0.001) as compared to the NPC group (MI change 0.064, *p* < 0.001; Figure 2G).

Changes of CFC significantly correlated with changes of VAS-L/VAS-A and modulus of tinnitus pitch change (Table 1). Changes of theta-gamma CFC between ACC and DLPFC as well as between ACC and AC were qualitatively similar as revealed by strong significant correlation between ACC theta—DLPFC gamma and ACC theta—AC gamma MI changes (*r* = 0.67, *p* < 0.01). These results together with absence of significant changes of the phase synchronization between AC and DLPFC (Tables 2, 3) indicate that the gamma amplitude in the AC and DLPFC may have been similarly modulated by the theta phase in the ACC.

**Table 1:** **Correlations between changes of MI and changes of the modulus of the tinnitus pitch change ratio, VAS-L/VAS-A**.

	**Modulus of relative pitch change**	**Relative VAS-L change**	**Relative VAS-A change**
MI change ACC theta—AC gamma	*r* = −0.41, *p* = 0.03	*r* = −0.44, *p* = 0.01	*r* = 0.78, *p* = 0.04
MI change ACC theta—DLPFC gamma	*r* = −0.40, *p* = 0.02	*r* = −0.41, *p* < 0.01	*r* = −0.49, *p* < 0.01
MI change ACC theta—DLPFC gamma	*r* = 0.66, *p* < 0.01	*r* = 0.31, *p* > 0.05	*r* = 0.32, *p* > 0.05

**Table 2:** **Coherence between selected regions for the selected frequency bands**.

	**AC-DLPFC**			**ACC-AC**			**DLPFC-ACC**		
	**Baseline**	**12 weeks**	**Change**	**Baseline**	**12 weeks**	**Change**	**Baseline**	**12 weeks**	**Change**
Delta/Theta	0.035	0.031	−0.001	0.035	0.039	0.004	0.056	0.047	−0.009
Alpha	0.045	0.043	−0.002	0.054	0.048	−0.006	0.032	0.029	−0.003
Gamma	0.047	0.036	−0.011^*^	0.087	0.079	−0.008	0.052	0.036	−0.016

The baseline and 12 weeks coherence and the change during the 12 weeks treatment period are shown for the bands where CFC interaction was found.

* p < 0.05, n = 32.

**Table 3:** **Phase synchronization between selected regions for the selected frequency bands**.

	**AC-DLPFC**			**ACC-AC**			**DLPFC-ACC**		
	**Baseline**	**12 weeks**	**Change**	**Baseline**	**12 weeks**	**Change**	**Baseline**	**12 weeks**	**Change**
Delta/Theta	0.15	0.16	0.01	0.20	0.19	−0.01	0.16	0.16	0.00
Alpha	0.16	0.20	0.04	0.16	0.17	0.02	0.16	0.20	0.04
Gamma	0.18	0.11	−0.07^*^	0.18	0.17	−0.02	0.18	0.12	−0.06

* p < 0.05, n = 32.

### Phase synchronization and coherence between ROIs

To further investigate the processes that lead to abnormal cross-frequency interactions in tinnitus patients and CFC changes after the CR therapy, we analyzed the PS and coherence at baseline and at 12 weeks as well as PS and coherence changes between baseline and 12 weeks for all subjects. We focused on the same frequency PS and coherence between pairs of ROIs, i.e., interareal interactions within delta, theta, alpha and gamma bands. The results of the phase synchronization and coherence analysis are shown in Tables 2, 3. Significant changes of the PS and coherence were observed only in the gamma frequency band between AC and DLPFC (Tables 2, 3). No significant correlation was found between the changes of the PS or coherence in any of the frequency bands and changes of CFC. What is noteworthy here is the fact that the PS and coherence changes between areas (e.g., between ACC and DLPFC) within modulating frequencies (delta, theta and alpha) that were involved in the intraareal cross-frequency modulation of the gamma band were not correlated with the CFC changes. This indicates that the observed phase-amplitude cross-frequency interaction between areas is not associated with within frequency PS between these areas and is a separate phenomenon.

## Discussion

Both animal (Eggermont and Komiya, 2000; Eggermont, 2003; Seki and Eggermont, 2003) and human data (Weisz et al., 2005; van der Loo et al., 2009; De Ridder et al., 2011b; Adjamian et al., 2012; Adamchic et al., 2014) demonstrate that tinnitus is associated with increased oscillatory activity in delta, theta and gamma bands and increased neuronal synchronization in the auditory cortex and that the more this increase of neuronal activity and synchronization is pronounced, the higher is the intensity of the perceived tinnitus. Furthermore, reduction of oscillatory power in the delta/theta and gamma frequency ranges and increase of the oscillatory power in the alpha band in this area is associated with reduction of tinnitus loudness (Kahlbrock and Weisz, 2008; De Ridder et al., 2011b; Adjamian et al., 2012; Tass et al., 2012; Adamchic et al., 2014). The thalamocortical dysrhythmia model provides an explanation for the emergence and persistence of such a pattern of oscillatory activity as a result of sensory differentiation (Llinas et al., 1999). Particularly this model suggests that gamma oscillations emerge together with an increase in slow wave activity (Llinas et al., 1999; Weisz et al., 2007). This notion is further confirmed and extended by the current study, which revealed that in tinnitus, aside from the described changes of oscillatory activity, there is also a more pronounced periodicity of amplitude variations of higher frequency (gamma) oscillations locked to certain phases of lower (delta/theta) frequency oscillations. Moreover reduction of initially increased cross-frequency interaction in the tinnitus patients was positively correlated with reduction of tinnitus after treatment with CR neuromodulation.

In our previous publications (Tass et al., 2012; Adamchic et al., 2014) and in the current study we revealed that in tinnitus both sensory and prefrontal areas are affected by similar abnormalities of oscillatory power and CFC. Moreover, the amount of the therapy induced pitch change was associated with the amount of changes of these abnormalities (Adamchic et al., 2012a). According to these results and the findings of previous studies (Weisz et al., 2005, 2006, 2007; Schlee et al., 2009; Vanneste et al., 2011; Adamchic et al., 2012a, 2014; Eggermont and Roberts, 2012; Tass et al., 2012), we can summarize that conscious tinnitus perception involves abnormalities in activity of auditory cortices together with abnormal processing across cortical circuits, including the prefrontal cortex. Moreover, the oscillatory activity in both the sensory and prefrontal regions may be modulated by other cortical areas in a tinnitus aversive network, specifically, the limbic system (Jastreboff, 1990; Rauschecker et al., 2010; De Ridder et al., 2011a, 2014). In such a network, coupled delta/theta-gamma activity may serve as an important mechanism for communication between these areas (Canolty et al., 2006).

In modeling studies it was shown that periodically firing or bursting neurons display the dynamics of limit cycle oscillators (Murray, 1989) which can be approximated by means of phase oscillators (Kuramoto, 1984; Hansel et al., 1993a,b). Mathematically it was shown that the coupling between neurons may cause mutual phase differences, frequency shifts, different types of synchronization patterns such as in-phase synchrony and cluster states (Tass, 1995, 1997). For strong enough coupling stable fixpoint-type states emerge, whereas for weaker coupling so-called running solutions emerge, where these different variables undergo variations in time (Murray, 1989; Guckenheimer and Holmes, 1990). On mesoscopic or macroscopic levels, assessed by local field potentials or MEG/EEG recordings, this translates to a variety of modulatory dynamical phenomena, such as power to power CFC, phase to phase CFC, phase to power CFC, phase to frequency CFC, power to frequency CFC, and frequency to frequency CFC (for review see Jirsa and Müller, 2013). Transient coupling between low and high frequencies was proposed to coordinate activity between task-relevant regions providing a mechanism for effective communication during cognitive, sensory and memory processing (Buzsaki and Draguhn, 2004; Canolty et al., 2006; Doesburg et al., 2012). Brain oscillations of different frequencies can interact within and between various brain areas, e.g., in the following ways: (i) amplitude modulation of a higher frequency oscillation by the phase of a slower rhythm, that is, phase-amplitude coupling (ii) phase modulation of a higher frequency oscillation by the phase of a slower rhythm, that is, phase-phase coupling (iii); amplitude modulation of a higher frequency oscillation by the amplitude of a slower rhythm that is, amplitude-amplitude coupling (Tass et al., 1998; Payne and Kounios, 2009; Canolty and Knight, 2010; Tort et al., 2010; Fell and Axmacher, 2011; Onslow et al., 2011). These types of CFC may occur independently, may have different mechanisms and can lead to different functional consequences (Fell and Axmacher, 2011). Phase–amplitude coupling was demonstrated in working memory tasks in which rats, while navigating through a T-maze, had to make decisions and thus recruit working or long-term memory (Tort et al., 2008). In humans phase–amplitude coupling between theta phase and gamma (20–40 Hz) frequency oscillations amplitude increased while retaining novel information in the working memory (Axmacher et al., 2010). Based on these findings it has been suggested that phase–amplitude coupling mechanisms in the limbic system and prefrontal cortex may support phase-dependent coding of objects (Fell and Axmacher, 2011).

Our study revealed that alpha oscillations exert cross-frequency influence on gamma activity. Alpha oscillations were proposed to exercise “pulsed” functional inhibition of irrelevant neuronal processing streams (Jensen and Mazaheri, 2010; Mazaheri and Jensen, 2010; Siegel et al., 2012). Accordingly, in the tinnitus network transient periods of alpha-entrainment from the ACC may pulse modulate gamma activity (Osipova et al., 2008) in the DLPFC as implied by the increase of alpha activity in the ACC, increase of phase-amplitude CFC (between the ACC and the DLPFC) and the simultaneous decrease of gamma power in the DLPFC with increase of tinnitus pitch change (Adamchic et al., 2012a). Thus, we can speculate that increase in the alpha activity in the ACC through its influence on an inhibitory control system of the dorsolateral prefrontal cortex can lead to an increase in the magnitude of inhibitory bouts on the DLPFC and break the local ongoing gamma activity (Medalla and Barbas, 2009; Jensen and Mazaheri, 2010; Mazaheri and Jensen, 2010). This notion would be in line with data showing that the ACC has strong influence on an inhibitory control system of the dorsolateral prefrontal cortex, more specifically BA 9, and can effectively enhance inhibition in that area (Medalla and Barbas, 2009). The stronger ACC alpha may lead to the shorter “duty-cycle” of gamma activity in the DLPFC providing graded inhibition of gamma oscillations by blocking it in a phasic manner, that is, by decreasing the time-window (duty-cycle) of processing (Jensen and Mazaheri, 2010). Reversing this, the reduction of alpha oscillations in patients with chronic tinnitus may lengthen the time-windows for gamma activity, thus, leading to increased gamma activity. Coupling of alpha oscillation with higher frequency, that is, gamma neural activity, may be ubiquitous throughout the cortex and thus throughout the tinnitus network (Palva et al., 2005, 2010; Palva and Palva, 2007; Osipova et al., 2008; Voytek et al., 2010). This notion is further supported by our finding of alpha-gamma CFC within the AC and the DLPFC.

Involvement of theta activity in tinnitus pathophysiology is also widely accepted (Llinas et al., 1999; De Ridder et al., 2011b). In our previous studies we have shown that tinnitus reduction by CR neuromodulation is related to a normalization of increased theta activity in the medial prefrontal cortex (Tass et al., 2012; Adamchic et al., 2014). The modulation of gamma-band synchronization by the theta rhythm was established in multiple studies (Bragin et al., 1995; Lakatos et al., 2005; Canolty et al., 2006). The phase of the theta oscillation may define the time window when transfer of information is most efficacious (Hasselmo et al., 2002a,b; Fell and Axmacher, 2011). Moreover, oscillations in the theta frequency range may be more efficacious for information transfer over longer distances in the brain than gamma phase synchronization (von Stein and Sarnthein, 2000; Hasselmo et al., 2002a,b; Buzsaki, 2009). Thus, within the tinnitus network theta activity may coordinate local assemblies that are synchronized in the gamma range. Our analysis revealed that the phase of theta activity in the ACC modulated the synchronization in the gamma band in both the AC and the DLPFC. Increased interaction between the DLPFC and sensory lower-order areas, that is, the AC (that is confirmed by the significant reduction of the phase synchronization between DLPFC and AC) during presence of aversive auditory pitch may serve the purpose to prioritize further processing of that stimulus as the most relevant (Zatorre et al., 1994; Fuster, 2008). Such modulation of oscillatory rhythms in both the AC and the DLPFC regions appears to be coordinated by the ACC, and more specifically by the theta rhythm. Thus, coordinated theta activity may establish long-range brain networks, in which information is processed by gamma activity, nested on this theta activity. The ACC may have an important role in regulation of neuronal circuitry for cognitive and sensory domains and can be identified as a distinctive region in the tinnitus affective network.

For the within-band interaction, we did not find consistent changes of phase synchronization or coherence between the investigated anatomical regions (i.e., between DLPFC and ACC and between ACC and AC) in the frequency bands that were involved in the phase-amplitude CFC, except reduction of the gamma PS and coherence between AC and DLPFC. In addition, the values of PS are, in general, small. This shows, on the one hand, that the observed CFC is not an epiphenomenon of an underlying single band synchronization between the two regions. On the other hand, because theoretically single-band synchronization and CFC between the same two regions can coexist, the phase-amplitude CFC shown here is an exclusively non-linear effect independent from the within-band synchronization in the frequency band involved in the phase-amplitude CFC between the two involved regions (Darvas et al., 2009; López-Azcárate et al., 2013). Furthermore, the independence between changes of CFC and single-band phase synchronization changes allow to speculate that the observed CFC in the tinnitus network may provide an information transfer between nodes of this network not realizable by simple single-band synchronization.

An alternative explanation for CFC without any apparent interareal phase synchronization in the e.g., theta band is that feed-forward inhibition rather than excitation may be the main contributor to the observed CFC. Specifically, phase locking to the modulating frequency in the modulated area may arise through modulation of pyramidal cells excitability mediated via local inhibitory networks (Tierney et al., 2004; Siapas et al., 2005). This may explain the modulation of auditory and prefrontal gamma activity by limbic theta activity even in the apparent absence of phase synchronous theta activity in the modulated regions (i.e., prefrontal and auditory cortices), and as a result absence of any apparent interareal phase synchronization. If the output of the cingulate cortex, also through polysynaptic connections, terminates not on the pyramidal prefrontal and auditory neurons but rather on interneurons, then generated rhythmic synaptic currents are unlikely to add to a microscopic field oscillation (Niedermeyer and Lopes da Silva, 1999; Siapas et al., 2005) because of rather random geometric configuration and small size of the interneurons and their dendrites. Thus, rather than hyperpolarizing the pyramidal cells, the cingulate input can rhythmically decrease pyramidal cells impedance, thus modulating their excitability (Niedermeyer and Lopes da Silva, 1999; Siapas et al., 2005). Another, more general explanation for the amplitude of a fast rhythm being modulated by the phase of a slow rhythm comes from a computational study showing that periodic input into generic networks of bursting neurons or networks of phase oscillators causes a quasiperiodic modulation of the amplitude of the macroscopic variable assessing the network's collective dynamics (i.e., the local field potential for the bursting neurons) over a wide range of the input intensity (see Figures 2, 7 in Popovych and Tass, 2011). Only at particularly high input intensities an n:m phase synchronization emerges (Popovych and Tass, 2011). Put otherwise, from a modeling standpoint this phenomenon actually occurs in a generic way for biologically realistic, i.e., weak to intermediate input intensities.

In our current study we found clear evidence of the influence of tinnitus severity and tinnitus pitch on theta-gamma and alpha-gamma coupling. More specifically, CFC between theta in the ACC and gamma in the DLPFC and the AC was increased in tinnitus patients and was reduced after reduction of tinnitus severity by CR therapy. Similarly tinnitus pitch reduction after therapy resulted in a decrease of the theta-gamma and increase of the alpha-gamma coupling. Thus, the strength of cross-frequency modulation of cognitive and sensory areas by limbic system may be related to the perceived subjective aversiveness of the tinnitus, which in turn depends on both loudness and pitch.

## Conclusion

The current study showed that reduction of tinnitus severity had a large impact on the cross-frequency interaction in the AC, the DLPFC as well as between the ACC and both the DLPFC and the AC. Even though the reduction of CFC was not solely reliant on the amount of tinnitus pitch change, the presence of pronounced tinnitus pitch change significantly increased changes in CFC. Modulation of the amplitude of high-frequency oscillations by the phase of oscillations in lower frequency ranges can coordinate tinnitus-relevant activity in distributed cortical areas providing a mechanism for effective communication between these areas during cognitive, memory, and auditory processing of the tinnitus, that is, orchestrating a widespread tinnitus-related gamma network. The strength of the interaction between cognitive, sensory areas and the limbic system may reflect both the tinnitus related distress and the subjective aversion to the perceived auditory pitch.

### Conflict of interest statement

Ilya Adamchic reports no commercial or financial relationships that could be construed as a potential conflict of interest. Berthold Langguth received honoraria and speakers' fee from ANM, Astra Zeneca, Autifony, Lundbeck, Merz, Magventure, Novartis, Pfizer and Servier, research funding from the Tinnitus Research Initiative, the German Research Foundation, the German Bundesministerium für Bildung und Forschung, the American Tinnitus Association, Astra Zeneca and Cerbomed, funding for equipment from Magventure and travel and accommodation payments from Medtronic, Lilly, Servier and Pfizer. Christian Hauptmann: employment Jülich Research Center & former employment ANM GmbH. Christian Hauptmann has received research funding from the European Community, the Federal Ministry of Education and Research (Germany), the Deutsche Forschungsgemeinschaft, the Helmholtz Association. Peter A. Tass: employment Jülich Research Center; worked with ANM GmbH (Cologne, Germany) to develop devices for CR neuromodulation in patients, shareholder of ANM GmbH. Peter A. Tass has received research funding from the European Community, the Federal Ministry of Education and Research (Germany), the Deutsche Forschungsgemeinschaft, the Helmholtz Association, Biomedical Primate, the Michael J. Fox Foundation.
